# Treating *Helicobacter pylori* and Recurrent *Clostridioides difficile* Coinfection: A Delicate Balance in Management and a Need for Guidelines

**DOI:** 10.14309/crj.0000000000001369

**Published:** 2024-06-03

**Authors:** Yazan Abboud, Benjamin Richter, Raj Malhotra, Sima Vossough-Teehan

**Affiliations:** 1 Department of Internal Medicine, Rutgers New Jersey Medical School, Newark, NJ; 2 Division of Gastroenterology and Hepatology, Rutgers New Jersey Medical School, Newark, NJ

**Keywords:** Helicobacter pylori, Clostridioides difficile, coinfection, outcomes

## Abstract

Treating *Helicobacter pylori* and *Clostridioides difficile* coinfection presents a challenging clinical dilemma. Treating *H. pylori* may increase the risk of *C. difficile*, and antibiotics generally have been shown to increase the risk of *C. difficile* infection/recurrence. While it may be reasonable to delay *H. pylori* treatment, this is especially challenging when there is an acute indication to treat *H. pylori* such as peptic ulceration or bleeding. There are no guidelines on the management of *H. pylori* and *C. difficile* coinfection. We report a patient who had *H. pylori* and recurrent *C. difficile* coinfection and suggest a management algorithm based on literature review and our institutional experience. Our patient received quadruple therapy for *H. pylori* along with vancomycin prophylaxis, taper, and a dose of bezlotoxumab and experienced good outcomes with resolution of his gastrointestinal bleeding and diarrhea.

## INTRODUCTION

Since the cytopathic involvement of *Clostridioides difficile* in pseudomembranous colitis was first reported in 1978, the incidence and severity of *C. difficile* infection (CDI) have been topics of active research.^1^ Complications of CDI can be severe including toxic megacolon, bowel perforation, and death, emphasizing the need for appropriate and timely management. Soon after the implications of CDI were reported, the role of Helicobacter Pylori (*H. pylori*) in gastroduodenal ulcers and gastric cancers was demonstrated in 1982, and it was identified as the most common bacterial infection in humans, affecting about 50% of the world's population.^2,3^ Complications of *H. pylori* infection include gastritis, peptic ulcer disease, and gastric cancers.

Despite how commonplace *C. difficile* and *H. pylori* infections are, a true coinfection rate of these bacteria is unknown. However, *C. difficile* colitis occurring as a complication of *H. pylori* eradication therapy has been reported in the literature.^4,5^ Treating *H. pylori* and *C. difficile* coinfection presents a challenging clinical dilemma. Patient outcomes in CDI after eradication therapy have ranged from total resolution of symptoms to fulminant colitis.^6^ While it may be reasonable to delay *H. pylori* treatment to avoid the risk of worsening CDI, this is especially challenging when there is an acute indication to treat *H. pylori* such as peptic ulceration or bleeding. Despite the scarcity of reported coinfections, an established treatment guideline is necessary for appropriate intervention to mitigate the risk of complications from either bacteria. We report a patient who had recurrent *C. difficile* and *H. pylori* coinfection and suggest an evidence-based management algorithm.

## CASE REPORT

**Figure 1.**
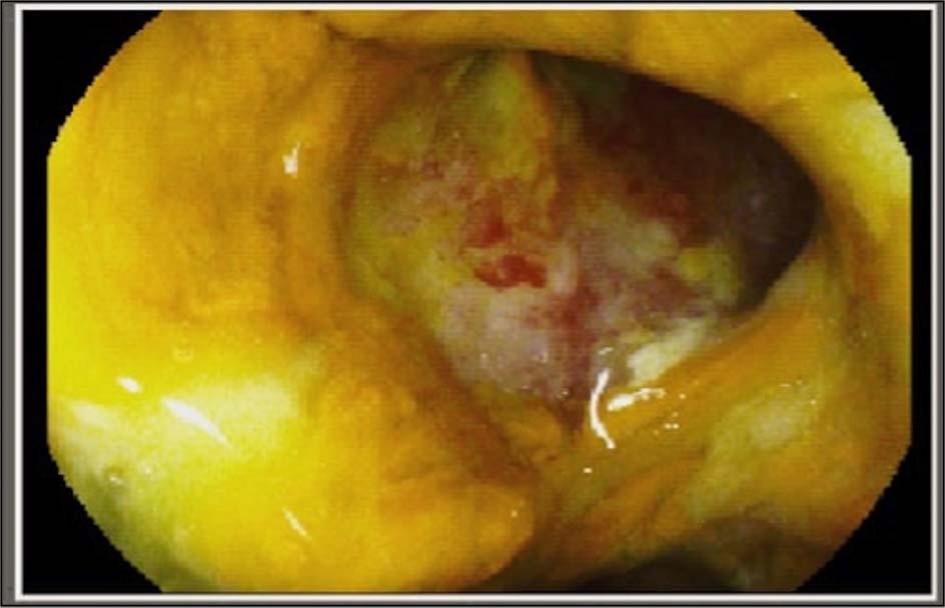
Endoscopic image of a giant at least 2 cm punched-out ulcer at the junction of the first and second portions of the duodenum.

An 80-year-old man with a history of perforated duodenal ulcer, surgically repaired 2 years ago, and recent *C. difficile* colitis with failure of treatment response to fidaxomicin and incomplete vancomycin course, presented with a 10-day history of 5 episodes of watery diarrhea per day. He was initially admitted to the intensive care unit for hypovolemic shock. *C. difficile* polymerase chain reaction (PCR) and toxin were positive, and he completed a 10-day vancomycin course. His symptoms improved, and repeat *C. difficile* PCR and toxin were negative; however, diarrhea later recurred with positive *C. difficile* PCR. Infectious stool studies were otherwise negative. He was diagnosed with recurrent *C. difficile* colitis and given bezlotoxumab and a vancomycin taper with plans to treat for 6–8 weeks. His hospital course was complicated by acute on chronic anemia requiring blood transfusions and prompting esophagogastroduodenoscopy, which revealed a 2 cm punched-out ulcer at the junction of the first and second portion of the duodenum with gastric biopsies showing *H. pylori* (Figure 1). Infectious disease was consulted, who advised delaying *H. pylori* treatment out of concern that antibiotics may worsen or prolong *C. difficile* colitis. The acute anemia subsequently worsened, which was deemed secondary to bleeding from the nonhealing duodenal ulcer, caused by untreated *H. pylori*. This prompted initiation of *H. pylori* quadrapole therapy with a 14-day course of tetracycline, metronidazole, bismuth, and omeprazole, and simultaneously switching vancomycin to prophylactic dosing (125 mg by mouth daily) while on *H. pylori* treatment instead of the previously recommended taper. After completing the 14-day *H. pylori* course, the vancomycin taper was resumed (125 mg by mouth 4 times a day for 14 days, then 125 mg by mouth twice a day for 7 days, then 125 mg by mouth daily for 7 days, and then 125 mg by mouth every other day for 14 days). The patient's diarrhea resolved without any complications, and his anemia stabilized.

## DISCUSSION

We report a rare case of an 80-year-old man with recurrent *C. difficile* and *H. pylori* coinfection who experienced persistent bleeding prompting esophagogastroduodenoscopy and eradication of *H. pylori* using quadruple therapy while administering prophylactic *C. difficile* treatment with vancomycin and subsequent vancomycin taper. We suggest an evidence-based algorithm for managing such cases, which showed good outcomes in our patient.

Literature discussing management strategies for *C. difficile* and *H. pylori* coinfection consists of a few case reports. In the first case report, a patient presented with abdominal pain and diarrhea; the patient tested positive for coinfection and was treated with vancomycin.^7^ However, after completion of treatment, he had recurrence CDI, for which he received fecal microbiota transplantation (FMT), observed for 1 month, and then underwent quadruple *H. pylori* eradication therapy with successful treatment of both infections. In the other case report, a patient with ulcerative colitis presented with weight loss and bloody diarrhea and tested positive for a coinfection. The decision was made to treat the *C. difficile* before *H. pylori* infection, to which the patient responded well.^8^ There are some limitations to the previously documented management strategies. In both reports, the patient did not experience acute complications from *H. pylori* that would otherwise prompt the need for acute management. Moreover, the unavailability of FMT for definitive treatment in recurrent *C. difficile* limits its utility. To date, FMT is inconsistently available in low-resource settings–the same settings in which many risk factors of *C. difficile* and *H. pylori* infections are prevalent.^9^

**Figure 2.**
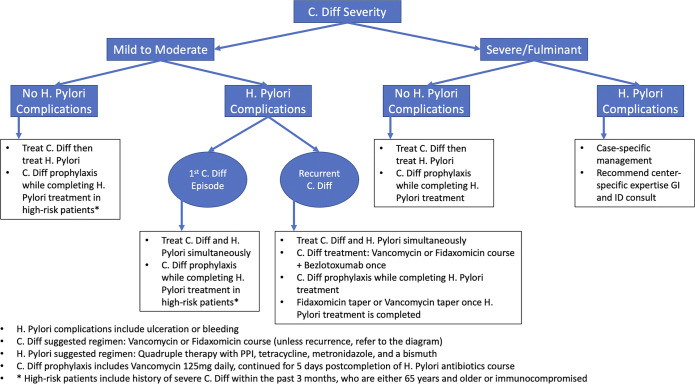
Suggested algorithm for treating *Clostridioides difficile* and *Helicobacter pylori* coinfection based on disease and patient-specific characteristics, literature review, and our institution experience.

According to our literature review and institutional experience, we propose a suggested algorithm for treating *C. difficile* and *H. pylori* coinfection based on disease and patient-specific characteristics (Figure 2). We based our approach on the Infectious Diseases Society of America (IDSA)/Society for Healthcare Epidemiology of America (SHEA) and American College of Gastroenterology guidelines.^10,11^ In our proposed coinfection treatment algorithm, we recommend quadruple therapy with metronidazole and tetracycline over triple therapy with clarithromycin and amoxicillin for *H. pylori* management. This regimen is preferred because amoxicillin and clarithromycin in triple therapy have a higher association with precipitating CDI compared with metronidazole and tetracycline in quadrapole therapy.^12–14^ Regarding oral vancomycin for primary or secondary prophylaxis of CDI, mixed data have been reported. One systematic review and meta-analysis demonstrated that oral vancomycin was ineffective for primary prevention of CDI but was effective in secondary prevention of recurrent CDI.^15^ Another systematic review and meta-analysis suggested that, in the setting of low-quality evidence, low-dose oral vancomycin prophylaxis can be used in select high-risk patients receiving systemic antibiotics associated with CDI.^16^ There is a need for large randomized clinical trials to clarify the utility of oral vancomycin prophylaxis for CDI. We also used bezlotoxumab which showed good outcomes aligning with prior literature of reducing risk of further recurrence in recurrent *C. difficile* cases.^17^

In conclusion, we suggest a management algorithm for treating *H. pylori* and *C. difficile* coinfection. This proposed algorithm stratifies CDI severity by mild to moderate and severe/fulminant (Figure 2). *H. pylori* infection is then classified as with or without complications. If no *H. pylori* complications are present, we recommend treating the CDI first and considering *C. difficile* prophylaxis while subsequently treating the *H. pylori* infection in patients at high risk of *C. difficile* or in patients with severe *C. difficile* If *H. pylori* complications are present with a mild-to-moderate CDI, we recommend *H. pylori* treatment and prophylactic *C. difficile* treatment administered concurrently in patients at high risk of *C. difficile* In severe *C. difficile* cases with complicated *H. pylori*, we recommend case-specific management in accordance with institutional experience. When there is an acute indication to treat *H. pylori*, it may be beneficial to favor quadruple over triple therapy. Prophylaxis vancomycin until completing *H. pylori* treatment, followed by a vancomycin taper, showed good outcomes in our case of recurrent *C. difficile*. Future studies are needed to externally validate our approach and establish guidelines for managing *H. pylori* and *C. difficile* coinfection to prevent complications and improve outcomes.

## DISCLOSURES

Author contributions: Y. Abboud: substantial contributions to the conception or design of the work; the acquisition, analysis, and interpretation of data for the work; and original drafting the work. B. Richter: design of the work; revising the work critically for important intellectual content. R. Malhorta: drafting parts of the work; revising the work critically for important intellectual content. S. Vossough-Teehan: substantial contributions to the conception or design of the work; analysis and interpretation of data for the work; and revising it critically for important intellectual content. S. Vossough-Teehan is the article guarantor.

Financial disclosure: None to report.

Informed consent was obtained for this case report.
